# Prevalence of diabetes mellitus among 80,193 gastrointestinal cancer patients in five European and three Asian countries

**DOI:** 10.1007/s00432-021-03861-7

**Published:** 2021-12-12

**Authors:** Christoph Roderburg, Sven H. Loosen, Laura Hoyer, Tom Luedde, Karel Kostev

**Affiliations:** 1 Clinic for Gastroenterology, Hepatology and Infectious Diseases, University Hospital Düsseldorf, Medical Faculty of Heinrich Heine University Düsseldorf, Moorenstraße 5, 40225 Düsseldorf, Germany; 2 IQVIA, Frankfurt, Germany

**Keywords:** Colorectal cancer, Diabetes, Prevalence, Europe, Asia

## Abstract

**Background:**

Diabetes mellitus (DM) has recently been associated with an increased incidence of such digestive tract malignancies as gastric or colorectal cancer. However, systematic data on the prevalence of DM among digestive tract cancer entities, especially in terms of geographic distributions, are lacking.

**Methods:**

We used the Oncology Dynamics database (IQVIA) to identify a total of 80,193 patients with gastrointestinal (GI) cancer (5845 esophagus, 20,806 stomach, 38,138 colon, and 15,414 rectum cancer patients) from eight European and Asian countries.

**Results:**

The overall prevalence of DM among all digestive tract cancer patients was 14.8% (11,866/80,193). In terms of cancer site, DM prevalence was highest in patients with colon (15.5%) or rectal (15.3%) cancer and lowest in patients with esophageal cancer (12.0%). Interestingly, we observed significant differences in DM prevalence between countries. Spain (27.8%, 31.3%) and South Korea (21.0%, 27.9%) had the highest prevalence of DM among gastric and colon cancer patients, while DM prevalence in esophageal (18.8%) and rectal (38.0%) cancer patients was highest in Germany.

**Conclusion:**

Our data revealed a high prevalence of DM among digestive tract cancer patients in Europe and Asia, and showed that DM prevalence varies among digestive tract cancer sites as well as countries.

## Introduction

Obesity, specifically abdominal obesity, and type 2 diabetes mellitus (DM) are frequently associated with metabolic abnormalities that may contribute to cancer progression, as recently demonstrated in animal models and in vitro approaches (Warr et al. 2018; Janssen 2021). In humans, the relationship between metabolic diseases and in particular DM and various cancers has been extensively studied. The National Health and Nutrition Examination Survey I (NHANES I) analyzed cancer risk in 14,407 men and women, and demonstrated that men with DM had an approximately 40% increased risk of developing cancer overall, with a particularly increased risk for developing colorectal cancer (Steenland et al. 1995). Both a meta-analysis of individual participant data from Europe and the USA and a literature-based meta-analysis demonstrated that prevalent diabetes was positively associated with colorectal cancer risk and inversely associated with prostate cancer risk in older Europeans and Americans (Ling et al. 2020; Amadou et al. 2021). Similarly, the Nurses' Health Study reported a 17% increased risk of breast cancer incidence in women with DM, compared with women without DM. In addition, a variety of other studies and meta-analyses comparing both incidence and mortality of malignancies in patients with DM and those in the general population have been conducted. A consistent effect has been seen on cancers of the digestive system, including colorectal cancer (Larsson et al. 2005), pancreatic cancer, and biliary tract cancer (Ren et al. 2011). Moreover, in men with DM, an increased incidence of esophageal cancer has been described (Huang et al. 2012). Finally, overall cancer mortality was suggested as being increased in patients with DM when compared to cancer patients without DM (Campbell et al. 2012; Barone et al. 2008), highlighting the tremendous impact of DM on cancer development and progression.

Although these and numerous other studies have demonstrated a clear association between cancer incidence and cancer-related mortality and digestive tract malignancies (Gallagher and LeRoith 2015), no comparative analyses regarding frequencies of DM in different countries or different health care systems are currently available. However, such data could help to gain a deeper understanding of the pathophysiology of these diseases and to better understand country-specific factors that lead to the development of such tumors with respect to DM. In the present study, we performed a retrospective cross-sectional analysis using data from IQVIA’s Oncology Dynamics (OD) database to compare the prevalence of DM among patients with GI cancers in eight different countries in Europe and Asia.

## Methods

### Database

This retrospective cross-sectional study is based on data from IQVIA’s Oncology Dynamics (OD) database (Zhao et al. 2012; Marchetti et al. 2017; Chambers et al. 2020). The source of this data is a cross-sectional, partially retrospective survey which collects anonymized patient data from a representative panel of oncologists. The OD survey collects fully anonymized patient-level data on drug-treated cancer cases in several countries worldwide. Data collection and reporting are conducted through a standardized online questionnaire where all items are mandatory. A reporting manual with precise instructions on filling out the questionnaire is provided to each respondent. Specific instructions are displayed through a ‘pop-up’ system throughout the survey to provide clear definitions for the desired variables. Physicians are also asked to enter factual information from patients’ medical records to avoid recall biases. Further tactics to ensure input accuracy include controlled code lists and multiple-choice questions, as well as interactive filters that limit non-applicable questions (e.g., items on cancer-specific biomarkers). Responses are immediately validated against previous answers and reference files; “unexpected value” messages are displayed to the participant if an irregular response is detected, prompting them to double-check their response. Physicians are instructed to report the most recent consecutive cases (up to 20 cases depending on the specialty) that they have treated during the last 7-day period to discourage selective case submission. After form submission, additional validations and trend checks are performed; anomalous values are discussed with the participants who submitted them and corrected as needed.

### Patient selection and study outcome

Survey data of all patients with one of four gastrointestinal tumors, including esophageal (ICD-10: C15), stomach (ICD-10: C16), colon (ICD-10: C18), and rectum (ICD-10: C20), that were submitted in the time between January 1, 2017 and March 31, 2021 were included in this study. Countries where data were available included Germany, France, the United Kingdom (UK), Spain, Italy, China, Korea, and Japan. The outcome of the study was the proportion of cancer patients with a documented diagnosis of diabetes mellitus (DM, diabetes including type 1 and type 2) (ICD-10: E10–E14) as a function of cancer type and country.

### Statistical analysis

We compared baseline characteristics for subjects with esophageal, stomach, colon, and rectum cancers using Chi-square tests for categorical variables (sex, facility, and country) and Wilcoxon tests for age. The prevalence of DM was calculated as the proportion of patients with DM on all patients and was shown for country by cancer types. To investigate the diabetes probability, a multivariable logistic regression model was fitted with DM (yes/no) as the dependent variable and cancer type and country as impact variables, adjusting for age, sex, and facility. The results of the regression analyses are presented as odds ratios (ORs) with 95% confidence intervals (CIs). *p* values lower than 0.05 were considered statistically significant. All analyses were performed using SAS 9.4 (SAS Institute, Cary, US).

## Results

### Baseline characteristics of study population

Overall, 80,193 people with GI cancer (5845 esophageal, 20,806 stomach, 38,138 colon, and 15,414 rectal cancer patients) documented by 2874 physicians were included in this study. The baseline characteristics of the study sample are displayed in Table 1. A very small but significant difference in age was observed (from 63.4 years in patients with rectal cancer to 65.2 years in colon cancer patients). The proportion of men was highest among esophageal cancer patients (76.5%) and lowest among patients with colon cancer (61.3%).

**Table 1:** Baseline characteristics of study patients

Variable	Cancer sites				*p* value
	Esophagus	Stomach	Colon	Rectum	
*N*	5845	20,806	38,138	15,404	
Age (mean, SD)	64.1 (9.2)	63.8 (11.1)	65.2 (10.9)	63.4 (10.9)	< 0.001
Males (%)	76.5	67.1	61.3	64.2	< 0.001
Facility					
Hospital	84.6	82.3	65.4	75.6	< 0.001
Oncologist in private practice	6.4	4.7	9.0	10.7	
Unknown	9.0	13.0	25.6	13.7	
Country					
Germany	15.2	8.4	12.9	14.8	< 0.001
France	12.3	4.7	10.9	8.3	< 0.001
Italy	9.0	12.9	25.6	13.6	< 0.001
Spain	9.5	5.5	11.7	13.0	< 0.001
UK	26.4	4.9	10.4	8.5	< 0.001
Japan	9.3	24.6	8.4	7.5	< 0.001
Korea	2.8	13.9	5.2	5.7	< 0.001
China	15.6	25.1	14.8	28.6	< 0.001

### Prevalence of diabetes mellitus among patients with GI cancer

The overall prevalence of diabetes mellitus (DM) was highest among patients with colon (15.5%) or rectal (15.3%) cancer and lowest in esophageal cancer patients (12.0%). Patients with stomach cancer had a DM prevalence of 14.0%. In a multivariate regression model, stomach cancer (OR 1.15, 95% CI 1.05–1.26, *p* = 0.003), colon cancer (OR 1.17, 95% CI 1.07–1.27, *p* < 0.001), and rectal cancer (OR 1.27, 95% CI 1.16–1.40, *p* < 0.001) were significantly associated with higher odds of DM compared to esophageal cancer as a reference group (Table 2).

**Table 2:** Association between GI cancer type, country, and the prevalence of diabetes mellitus in GI cancer patients (multivariable logistic regression model)

Variable	Average age of patients (mean, SD)	Proportion of patients with DM (%)	OR (95% CI)^a^	*p* value
Cancer type				
Esophagus	64.1 (9.2)	12.0	Reference	
Stomach	63.8 (11.1)	14.0	1.15 (1.05–1.26)	0.003
Colon	64.9 (11.0)	15.5	1.17 (1.07–1.27)	< 0.001
Rectum	63.0 (11.1)	15.3	1.27 (1.16–1.40)	< 0.001
Country				
Germany	65.1 (10.2)	18.3	1.65 (1.48–1.83)	< 0.001
France	65.6 (10.0)	16.3	1.44 (1.32–1.56)	< 0.001
Italy	65.8 (10.6)	14.8	1.24 (1.15–1.33)	< 0.001
Spain	65.0 (10.9)	21.4	2.02 (1.87–2.19)	< 0.001
UK	63.4 (11.2)	10.0	0.91 (0.83–1.00)	0.052
Japan	68.3 (9.7)	15.2	1.15 (1.07–1.25)	< 0.001
Korea	62.4 (10.9)	18.6	1.99 (1.83–2.17)	< 0.001
China	59.4 (10.7)	9.3	Reference	

^a^Multivariable logistic regression model adjusted for country, age, sex, and facility type

### Geographical variation of diabetes mellitus prevalence

**Fig. 1:**
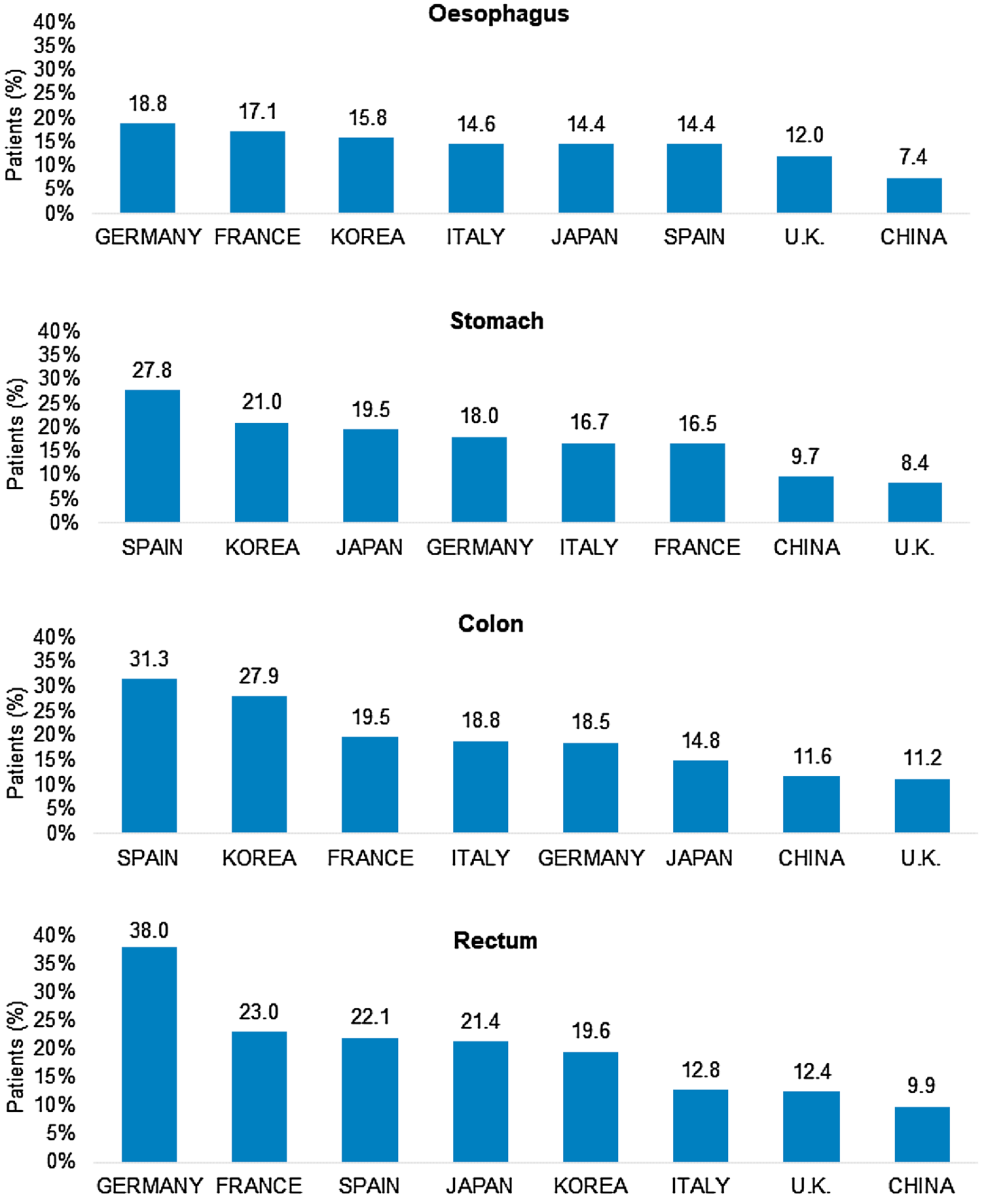
Prevalence of diabetes mellitus among patients with GI cancers in eight countries in Europe and Asia

Figure 1 shows the prevalence of diabetes mellitus (DM) in GI cancer patients. Among esophageal cancer patients, DM prevalence was highest in Germany (18.8%), followed by France (17.1%) and Korea (15.8%). Among stomach cancer patients, the countries with the highest prevalence of DM were Spain (27.8%), Korea (21.0%), and Japan (19.5%). In terms of colon cancer patients, Spain (31.3%) and Korea (27.9%) had DM prevalence rates that were much higher compared to other countries. Finally, we identified Germany as the country with the highest prevalence of DM (38%) in patients with rectal cancer. Interestingly, the proportion of DM for all cancer sites was lowest among patients in the UK and China. In the multivariate regression analysis, the odds for DM were highest in Spain (OR 2.02, 95% CI 1.87–2.19, *p* < 0.001), followed by Korea (OR 1.99, 95% CI 1.83–2.17, *p* < 0.001), Germany (OR 1.65, 95% CI 1.48–1.83, *p* < 0.001), France (OR 1.44, 95% CI 1.32–1.56, *p* < 0.001), Italy (OR 1.24, 95% CI 1.15–1.33, *p* < 0.001), and Japan (OR 1.15, 95% CI 1.07–1.25, *p* < 0.001), when compared to China as the country with the lowest DM prevalence.

## Discussion

By analyzing the prevalence of diabetes mellitus (DM) in patients with digestive tract cancers from eight different countries, we demonstrated that the distribution of DM is not only heterogeneous between different tumor entities but also between different countries. Such data add to the existing literature, suggesting that DM is an important metabolic risk factor for the development of cancer in humans.

Gastrointestinal cancers are among the most common tumor entities worldwide and have continuously rising incidence and prevalence rates (Xie et al. 2021). Similarly, DM represents a common disease and is associated with significant morbidity and mortality (Schacter and Leslie 2021). DM and cancer share a variety of pathophysiological features and risk factors, such as obesity and Western-style diet. Therefore, we used the IQVIA oncology dynamics (OD) database, which features clinical data from > 80,000 patients with digestive tract cancers, to analyze potential associations between these cancers and DM in eight different countries representative of many Western and Asiatic societies in terms of sociodemographic and medical characteristics. Regarding specific tumor entities, we observed the highest rates of DM in patients with rectal cancer, followed by colon, stomach, and esophageal cancers. Notably, the distribution of DM was very heterogeneous between the different countries. Overall, European countries such as Spain, Germany, and France displayed higher rates of DM among cancer patients compared to Asian countries such as Korea and China. This finding may reflect the different distribution of metabolic diseases, including obesity and DM, in the different countries worldwide, but it may also reflect the different relevance of DM as a risk factor in the different countries. It seems likely that modifying factors such as specific dietary habits like eating very spicy foods or diets rich in meat may modify the role of DM in the pathophysiology of the different cancers. Similarly, it was shown that the cancer-promoting effect of DM varies between male and female patients and different cancer sites (e.g., digestive tract vs. breast vs. urological cancers) (Suh and Kim 2019). Therefore, our data may help to unravel the differential role of DM in the pathophysiology of cancer and should trigger further research in this context.

Beyond these statistical associations, data from murine models have established DM as a strong risk factor for developing “any-site” cancer. On a molecular level, results from in vitro and in vivo studies have proposed different mechanisms regarding how DM may act as a driver of cancer development (Zhang et al. 2021). Most importantly, it was shown that excess insulin, a consequence of unhealthy diets and lifestyles, may promote inflammation and thereby have cancer-promoting effects (Orliaguet et al. 2020; Johnson and Olefsky 2013; DeFronzo et al. 2015). Furthermore, insulin is a powerful mitogen and survival factor for virtually all cell types, providing a potential mechanism for how diabetes may be associated with cancer (Zhang et al. 2021; Beith et al. 2008; Johnson et al. 2006).

The difference between prevalence of DM among GI cancers in different countries can be correlated with the prevalence of DM among the overall population in these countries. For example, the prevalence of DM in Germany was 9.7% in 2010 (Johnson et al. 2006) and in China 10.9% in 2013 (Tamayo et al. 2016), whereby both studies used different methods and definitions.

The main strength of the present study is the use of data from a large number of patients from various countries, enabling a better understanding of intercountry variation. However, our study is subject to several limitations. First, the original questionnaire was not designed for specific research purposes. Second, missing variables such as genetic factors and socioeconomic status are further limitations. Third, due to the cross-sectional design, no information was available on the order of events to enable us to identify whether cancer or diabetes was diagnosed first. Fourth, no stratification in type 1 and type 2 diabetes diagnoses was possible. Fifth, no causal relationships can be estimated in studies like this, only associations. Finally, as the database used is an oncology database, we could not investigate the prevalence of diabetes among individuals without cancer. Although the database has been used for many different studies and its suitability for research purposes in various clinical analyses has been demonstrated (Zhao et al. 2012; Marchetti et al. 2017; Chambers et al. 2020), we cannot rule out potential data quality differences between countries.
